# Da Vinci Single-Port Robotic Surgery in Europe: Where Do We Stand? A Systematic Review

**DOI:** 10.3390/jcm14238317

**Published:** 2025-11-23

**Authors:** Carlo Maria Scornajenghi, Beatrice Conti, Valerio Santarelli, Valentina Brunelli, Martina Moriconi, Roberto Acanfora, Giulio Bevilacqua, Giovanni Di Lascio, Giorgio Franco, Stefano Salciccia, Alessandro Sciarra, Giovanni Battista Di Pierro

**Affiliations:** Department of Maternal-Infant and Urological Sciences, Sapienza Rome University, Policlinico Umberto I, 00161 Rome, Italy; carlomaria.scornajenghi@uniroma1.it (C.M.S.);

**Keywords:** da Vinci Single-Port System, robot-assisted surgery, minimally invasive surgery, European single-port surgery experience

## Abstract

**Background/Objectives**: The da Vinci Single-Port (SP) system represents a recent evolution in robotic-assisted surgery, offering enhanced articulation and access through a single incision. The SP system was approved by the European Medicine Agency (EMA) in January 2024. **Methods**: This review synthesizes current clinical evidence on the feasibility, safety, and versatility of SP-assisted procedures across multiple surgical specialties to date based on a comprehensive literature search conducted through major databases (MEDLINE, EMBASE, and Cochrane) according to PRISMA and PICOS guidelines. **Results**: A total of 14 studies were included, highlighting that the SP platform has been successfully adopted in complex procedures such as cervical esophagectomy, radical prostatectomy, nephrectomy, gynecologic procedures, and wall surgery. Across indications, the SP approach is associated with reduced blood loss, shorter hospital stays, and low complication rates. On the other hand, limitations include restricted working space and the steep learning curve. **Conclusions**: Overall, the da Vinci SP platform expands the scope of minimally invasive surgery, but European centers lag behind international trends, particularly when compared to new, less-invasive techniques adopted in high-volume SP centers in the US. Urology remains the main field of application.

## 1. Introduction

Minimally invasive surgery is one of the major steps forward in modern surgical practice. Its goal is to reduce recovery time and morbidity while maintaining oncological and functional outcomes. In the last decades, robotic technology has improved surgical precision and visualization across different specialties.

During this evolution, LaparoEndoscopic Single-Site Surgery (LESS) was introduced in order to minimize surgical invasiveness, by allowing many instruments to be inserted through a single incision. Even if LESS can provide clear cosmetic advantages and less postoperative pain, its adoption has been limited by the difficulties in instrument triangulation and collision between tools [1,2,3].

To combine the precise vision and dexterity of Robotic-Assisted Surgery (RAS) and the reduced invasiveness of single-site surgery, a new platform was subsequently developed, the single-port (SP) robotic platform, for single incision robotic surgery through a multichannel access port. This system combines a fully articulating 3D high-definition camera with flexible wristed instruments, allowing safe and effective surgery even in confined spaces. Despite its relatively recent introduction into surgical practice in 2018, the SP surgical system has already demonstrated its advantages across different surgical specialties, mainly represented by faster hospital discharge, minor postoperative pain, and excellent cosmetic results, without compromising operative efficacy compared with the traditional multi-port (MP) platform.

After its regulatory approval in Europe in 2024, SP Robotic Surgery (SPRS) has seen a widespread diffusion through European centers. However, the current number of evidence remains limited due to recent introduction. A study conducted by the Single-Port Advanced Research Consortium (SPARC) Collaborative Group among European robotic surgeons highlighted a growing interest in adopting single-port systems, even though most respondents had no single-site experience (61% of the participants perceived advantages were reduced invasiveness, 53% easier access to retroperitoneal or extraperitoneal spaces, improved cosmetic outcomes by 44%, and lower postoperative pain in 44%) [4].

In US experiences, these advantages have often translated into a higher rate of complex procedures performed in an outpatient setting. The SP system is not, however, without drawbacks. Particularly, it has been criticized for the narrower workspace and more difficult instrument handling, which could potentially cause a longer and steeper learning curve [5].

This review is about the present European experience with SP surgery in different surgical techniques through various specialties, like nephroureterectomy, partial nephrectomy, lymphadenectomy for gynecologic and urologic malignancies, esophagectomy, and transoral tonsillectomy.

The focus of this work is to systematically summarize and narratively discuss data on feasibility, safety, perioperative outcomes, and potential benefits in terms of reduced invasiveness and improved postoperative recovery in the SP approach across surgical specialties and briefly compare the European data with those from US experiences.

## 2. Materials and Methods

This review was written according to the Preferred Reporting Items for Systematic Reviews and Meta-Analyses (PRISMA) guidelines [6] and according to the PICOS model framework [7].

The study population included adult patients who underwent robotic surgical procedures using the da Vinci SP platform in European centers regardless of surgical indication. A descriptive comparison with multi-port or laparoscopic approaches was made when available. Feasibility, safety, perioperative outcomes, complication and conversion rates, length of hospital stay, and cosmetic results were assessed. Eligible study designs encompassed case reports, case series, technical notes, or observational studies published between January 2024 and September 2025 in English.

No preliminary protocol was registered. An extensive and independent search was performed by two authors (C.M.S. and B.C.) on major scientific databases such as MEDLINE, EMBASE, and Cochrane. The search terminology was the following: (“Robotic Surgical Procedures” [MeSH] OR “robot-assisted surgery” OR “robotic surgery” OR “robot-assisted laparoscopic surgery” OR “robotic-assisted procedure”) AND (“single port” OR “single-port” OR “single incision” OR “single site”) to identify studies reporting the use of robotic single-port surgery in any surgical specialty.

The initial search found 1325 articles. To focus on the European experience following the EMA approval of the da Vinci SP^®^ system (Intuitive Surgical Inc., Sunnyvale, CA, USA), we considered only studies published between January 2024 and 30 September 2025. After using this filter, 365 studies remained.

Titles and abstracts were independently screened to identify works describing clinical applications of the da Vinci SP platform in European centers. Case reports, case series, and retrospective studies were included. Exclusion criteria were non-European populations, non-robotic single-port or conventional LESS techniques, dry lab, or cadaveric session.

**Figure 1:**
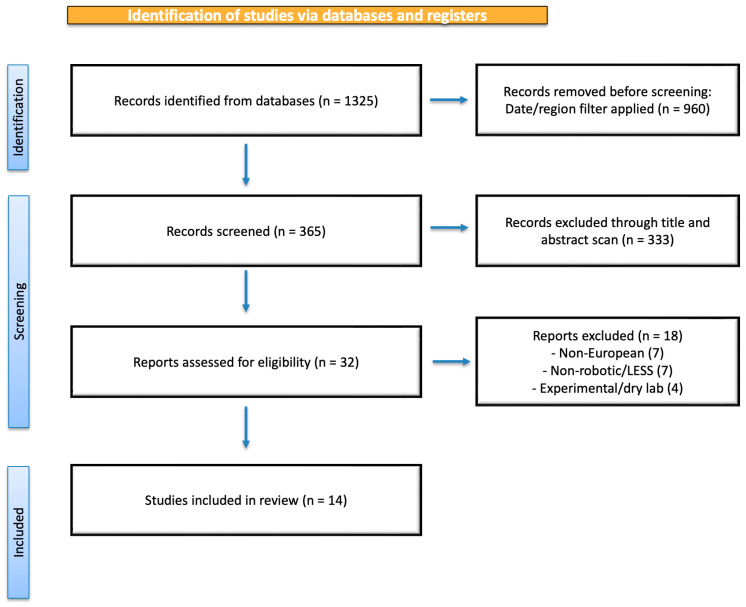
Flow diagram of selection of eligible studies.

After full-text screening, 14 studies were selected (Figure 1) for the final analysis procedures such as nephroureterectomy, partial nephrectomy, pelvic and retroperitoneal lymphadenectomy in gynecologic and urologic malignancies, esophagectomy, and transoral tonsillectomy. For each study, data were selected on study design, surgical indications, operative technique, surgical technique, patient position, robotic configuration, needs of accessory trocars, perioperative outcomes, complications, and postoperative recovery when available.

Due to the heterogeny of the studies, the narrative synthesis according to PRISMA 2020 recommendation was adopted and results were organized by surgical area to better show technical variances and outcome differences.

Most of the studies have a descriptive observational method (case reports and small series); for this reason, there is no formal risk of bias. The main methodological limitations (heterogeneity of samples, lack of a control group, and small sample sizes) were considered in the qualitative discussion of the results.

The main objective was to provide an integrated and comparative vision of SP experiences in Europe.

## 3. Results

A total of 14 studies were included in the final qualitative synthesis, encompassing 5 urological, 4 gynecologic, 4 general surgery, and 1 otolaryngologic report. Study characteristics are summarized in Table 1 and the selection process is detailed in Figure 1.

### 3.1. Urological Applications

Urological studies reported the application of SPRS for oncological reasons which represented the most common field in the European SP experiences in the identified studies.

The first European case series on SP Robot Assisted Nephroureterectomy (RANU) for Upper Tract Urothelial Carcinoma (UTUC) was published by Izzo et al. [8] in 2025. Authors reported the results of eight patients undergoing SP RANU via a supine anterior retroperitoneal approach, with a small bump placed under the flank, and left arm remained open as simplified by Pellegrino et al. [22]. This approach allowed the complete and precise dissection from kidney to bladder cuff without a re-docking with lower anterior access (LAA) [23], and only a single 6 cm incision, approximately at McBurney point (one-third lateral of the line between the umbilicus and the superior anterior iliac spine). The patients were discharged the day after with no complication, minimizing postoperative pain, showing feasibility and safety of supine position procedures. The configuration adopted (“camera-below” orientation) and a simplified arm layout (bipolar left, scissors right, and Cadiere grasper accessory), and enhanced maneuverability in the confined retroperitoneal workspace. The combined use of bipolar Maryland, monopolar forceps, and Cadiere grasper allowed a precise dissection of renal hilum and Ureter with minimum blood loss and no need for conversion. Postoperative pain was minimal with fast recovery and early discharge. No significant limitations were observed. Particular attention should be placed on severely obese patients, those with a history of recurrent UTIs and case requiring extensive lymph node dissection, considering the reduced working space or the presence of adhesions in an already limited operative field.

Similarly, Altez-Fernandez et al. [9] in 2025 reported a case series about retroperitoneal SP robotic-assisted partial nephrectomy (SP RAPN). Despite this series adopting the da Vinci Xi^®^ system (Intuitive Surgical Inc., Sunnyvale, CA, USA) in SP approach, it represents the first case series of SPRS in Europe. The study confirmed that surgery was technically feasible and safe, in a lateral decubitus position with no conversions, minimal blood loss, and cosmetical satisfaction with short length of hospital stay. Retroperitoneal space was developed under direct vision using a dissecting balloon (Applied Medical, Rancho Santa Margarita, CA, USA), followed by the attachment of the GelPOINT^®^ system (Applied Medical, Rancho Santa Margarita, CA, USA). Only three robotic arms were used with a 30° camera faced up. All procedures were on a clamp with less than 20 min warm ischemia and no additional ports were used. All patients had no recurrence at follow-up with only 4 cm scar. However, a positive surgical margin was reported in one case, and the bigger lesion was 4.2 cm. Further studies and native SP System adoption should be performed to evaluate outcomes in patients with larger tumors and to assess margin status more comprehensively.

Perdonà et al. [10,11] in 2025 expanded SP application to Inguinal lymph node dissection (ILND) for penile cancer and unilateral retroperitoneal lymph node dissection (RPLND) for testicular cancer.

Regarding ILND, a preoperative 3D Reconstruction allowed detailed visualization of inguinal structures thanks to the Tilepro feature. A 3 cm transversal skin incision was performed bilaterally at the distal point of Scarpa’s Triangle. Superficial and deep inguinal nodes were dissected. Fast recovery with fewer incisions when compared to MP surgery was noted. Reported limitations include longer operative time and steeper learning curve. Re-docking was necessary to perform bilateral dissection and in case of the need to perform pelvic lymph nodes dissection, a third incision with consequent repositioning of the SP system would be required, thereby undermining the concept of true single-port surgery.

SP-rRPLND through LAA with a 2.5 cm McBurney incision was performed for retroperitoneal access. Instrument configuration followed a “Camera below” setting, and confirmed the feasibility of single-port techniques even in post-chemotherapy fields after orchiectomy. Dissection from the aortic bifurcation to the renal hilum was performed, preserving vascular structures. Operative times were under 80 min and had uneventful recovery.

Both studies were video case reports and need further data to better achieve their outcomes.

Regarding ILND, Brassetti et al. [12], in 2025, developed a Single-Port Antegrade Robotic Lymphadenectomy (SPARL) that overcame the limitations previously stated for the Perdonà technique, where an umbilical incision was made with patients in supine position with 15° Trendelenburg tilt. Subcutaneous antegrade dissection allowed the retrieval of inguinal LND and when indicated, pelvic LND with only one incision, and no additional ports were needed. Monopolar curved scissors were used in the dominant hand, bipolar forceps in the other one, and Cadiere forceps as third arm in case of the need for a nasogastric 16-Ch tube used as a suction device and smoke evacuation in the absence of a Remotely Operated Suction Irrigation System (ROSI) (Vascular Technology Incorporated, Nashua, NH, USA). In case of Inguinal LND, a peritoneal incision was made lateral to the obliterated umbilical to access Retzius space with a lateral position. This technique was reported on 10 patients and showed no intraoperative complications or conversion and fast discharge with late drain removal 25 days median (IQR:18–34) to reduce risk of symptomatic Lymphocele. The median operative time was 197 min and blood loss was negligible. No conversion reported. They also reported minor complications that were managed conservatively. The antegrade perspective provided a natural dissection trajectory, improving ergonomics and facilitating lymph node retrieval with minimal morbidity. By eliminating the need for multiple inguinal incisions, SPARL may enhance cosmesis while reducing the risk of surgical site infections and wound dehiscence.

### 3.2. Expansion Beyond Urology

#### 3.2.1. Gynecology

Chauleur et al. [13] showed a single case of SP robotic para-aortic lymphadenectomy (pre-, inter-, and latero-aortic) in under one hour for previous cervix carcinoma. A supine approach was used with infraumbilical access through an incision of 2.5 cm, and accessory trocar was placed for peritoneal retraction and smoke evacuation pre-emptively. Retroperitoneal space was dissected manually. Arms configuration was endoscope in a down position, with monopolar scissors in the right hand, bipolar forceps in the left, and Cadiere forceps on the top. A total of 17 para-aortic lymph nodes were dissected, and no drain tube was left. Same-day discharge and excellent cosmetic outcomes were achieved, demonstrating the potential of SPRS in complex gynecologic oncology. Limitations are that there was only one video case report without sufficient data on postoperative outcomes.

Vizza et al. [14] reported a series of robotic single-port hysterectomies (R-SPH) in 25 patients with low-risk endometrial cancer. Procedures were performed in dorsal lithotomy with steep Trendelenburg through a 2.5 cm umbilical incision. The endoscope was in the upper configuration; channel number 1 holds a Cadiere forceps; channel number 2, a Maryland bipolar instrument; and channel number 3, monopolar scissors (MCS). Hysterectomies and lymphadenectomy were performed. Safety was assessed by monitoring intraoperative and postoperative complications. Intraoperative parameters included conversion to open surgery, need for transfusion, and any robot-related technical issues. All operations were successfully completed with no conversions or intraoperative complications. Blood loss was minimal (median 67 mL), and median hospital stay was 3 days. Median operative time was 117 min with a decreasing time through the length of the study, suggesting a learning curve effect consistent with other published series worldwide.

In another retrospective, propensity-matched study, Vizza et al. [15] compared perioperative and short-term outcomes between R-SPH and robotic-multi-port hysterectomy (R-MPH) for staging early-stage endometrial cancer. The study included 354 women who underwent robotic hysterectomy, bilateral salpingo-oophorectomy, and nodal dissection. One hundred patients with comparable baseline characteristics were selected and matched in a 1:1 ratio (50 R-SPH and 50 R-MPH). Operative performance metrics and perioperative outcome were compared as follows: median operative time (120 vs. 115 min, *p* = 0.367), estimated blood loss (10 vs. 15 mL, *p* = 0.317), and hospital stay (median 3 days, *p* = 0.269) was comparable between groups. SP cases exhibited significantly shorter docking times (6 vs. 11 min, *p* < 0.001). No major complications nor intraoperative conversions were observed, Ninety-day complication rates were similar (12% SP vs. 8% MP, *p* = 0.740). This study represents the first, despite being retrospective, European comparative case between SP and MP systems, enlightening that the da Vinci SP^®^ system is safe and feasible for endometrial cancer staging, providing ergonomic and cosmetic benefits without compromising safety or efficiency.

Chiofalo et al. [16] reported seven patients retrospectively who underwent single-port for staging for epithelial ovarian cancer between September 2024 and March 2025 at the Regina Elena National Cancer Institute in Rome via a trans-umbilical route with a 3 cm vertical incision. The robot was docked from the patient’s left side. Maryland bipolar, monopolar scissors, and tenaculum were used and uterine manipulators were employed. Median hospital stay was 3 days. Performed procedures included hysterectomy and in the most advanced case, omentectomy. The system’s flexible architecture allowed deep pelvic access and precise lymph node dissection while maintaining minimal invasiveness. No blood transfusion nor grade ≥ II complications were reported.

#### 3.2.2. General Surgery

SPR cholecystectomy demonstrated safety, efficiency, and rapid postoperative recovery with minimal invasiveness in the Vicente et al. [17] case. Via a trans-umbelical 2.5 cm incision made after induced pneumoperitoneum through Verres needle, the SP is introduced in the abdomen. The 0° endoscope was used with monopolar scissors and bipolar forceps. The operative time was 38 min, and shortcomings were the inability to use advanced dissection tools such as Vessel Sealer, and the steeper learning curve. Overall, the system has demonstrated excellent feasibility, safety, and efficiency in robotic-assisted cholecystectomy.

The SP approach has also been successfully applied to cervical and subcostal esophagectomy by Hadzijusufovic et al. [18,19], achieving precise mediastinal dissection, reduced postoperative pain, and faster patient mobilization. In subcostal access, a 12 mm trocar was placed for thoracoscopy in addition of a 4 cm incision for SP access. Fenestrated bipolar forceps in position 1, a retractor in position 2, and scissors in position 3 were used. A 0-degree camera was placed in a “cobra” position. After lung collapsing, a total esophagectomy can be safely performed, preserving the thoracic duct. Lymph node dissection was feasible (tracheal bifurcation and inferior pre-trachealis’s one). The reconstruction phase was carried out after de-docking. No data about timing and perioperative outcome were stated.

The trans-cervical access involves previous MP abdominal procedure plus a 3 cm transverse incision on the neck where SP was docked. We used fenestrated bipolar forceps on position 1, a retractor on position 2, and Maryland bipolar forceps on position 3. A 30-degree camera is placed in an upward “cobra” position. Carinal lymph nodes dissection was conducted easily and specimens were carried trans-mediastinally trough a preformed channel in the abdominal previous phase after the de-docking. The median time was 5:13 min. Considering the surgical anatomy of the esophagus, noteworthy advantages of this method include controlled dissection of high esophageal tumors, superior left recurrent lymph node dissection, and reduced pulmonary complications attributed to complete extrapulmonary dissection. These results indicate that SPRS enhances surgical precision and ergonomics while minimizing tissue trauma, supporting its potential integration into routine general upper tract gastrointestinal surgery practice, even though only one patient per each procedure was reported.

Another case by Hipp et al. [20] reported a novel access in abdominal wall surgery. They performed SPr Extended Extraperitoneal Plasty (eTEP) sublay herniotomy for ventral hernia, giving a well-explained step-by-step procedure. In total, two patients were discussed with no complications nor hernia recurrence. The patients were positioned supine with a 15° Trendelenburg tilt; vertical incision were made lateral to retrorectus space and with a small space left from the costal margin. Surgical steps were the development of one-sided retrorectus space and port placement, cross-over of the midline (ideally) above the hernia defect anterior to the falciform ligament, connection of both retrorectus spaces, defect closure and restoration of the Linea alba, and mesh placement. Median operative time was around 200 min, and drainage was removed on postoperative day (POD) III. The main advantage was perfect ergonomics for suturing midline hernia defect. No complications or conversion were observed. Future evaluation of the technique is necessary to provide further safety and long-term efficacy results.

#### 3.2.3. Ear, Nose, and Throat

Transoral robotic surgery (TORS) was shown to be feasible by Seifen et al. [21], performing a single-case tonsillectomy. The patient was placed in supine position and the patient’s head was carefully reclined. An endoscope and two articulating instruments were inserted as follows: one monopolar cautery instrument, and one Maryland bipolar forceps. The docking time was 8 min, and operation was completed in 37 min. No significant intraoperative events were observed underlying the strength of SP surgery through narrow cavities. No compliances were observed.

Across all included European studies (n = 14), the da Vinci SPS demonstrated consistent technical feasibility, excellent cosmetic output, and favorable ergonomics in all disciplines evaluated. The small skin incision and supine or lateral decubitus allowed optimal organ access with negligible blood loss and a mean time of operation from 30 to 200 min. Discharge was reported same day or ≤2-day in 70% of cases.

Each author described their own settings of robotic arms and camera disposition and variable operating time based on surgical experiences with this new technique resumed in Table 1.

Overall, the available evidence consistently demonstrates the technical feasibility of SP robotic surgery across multiple specialties, with favorable perioperative outcomes. However, the evidence remains preliminary and primarily descriptive, limiting the strength of clinical conclusions.

## 4. Discussion

The introduction of the SP robotic system has been the most important surgical hardware innovation of the last seven years. Since FDA approval in 2018, its popularity and diffusion have rapidly spread nationwide across the USA. Multiple high-volume centers in the US have published consistent data confirming the safety, feasibility, perioperative advantages, and tailored their main field of interest to minimally invasive oncological, reconstructive, and natural cavities surgery [24,25,26,27].

The SP system has demonstrated faster docking time and lower dependency on the bed-side assistant. Particularly, the valveless AirSeal^®^ valveless insufflation system (ConMed, Utica, NY, USA) can be directly connected to an assistant port positioned through the dedicated space of the access port, without the need for additional incisions. The ROSI Remotely Operated Suction Irrigation System (ROSI; Vascular Technology Inc., Nashua, NH, USA), can be easily inserted through the aforementioned access port and dropped into the surgical field, to be manipulated directly by the console surgeon with robotic instruments. The connected foot switch allows the robotic surgeon to have independent control over the activation and deactivation of suction and irrigation [28]. The system is not yet approved for use in Europe.

In urology, large-scale reviews such as Omidele et al. [29] summarized more than forty studies, assessing feasibility across all genitourinary cancers and reporting radical prostatectomy performed through different approaches (transvesical and retzius-sparing), cystectomy demolition phase, and some limited indications for intracorporeal reconstruction phase, although the single incision allows easy extracorporeal urinary diversion without any other unaesthetic scars.

Similarly, Valenzi et al. [30] showed that single-port radical prostatectomy in elderly patients significantly reduced postoperative complications and length of stay compared with the MP system, supporting its safety even in frail populationswith similar outcome to the SP RAPN by Santarelli et al. [31]. In another study, Valenzi et al. [32] underly advantages of the SP system comparing pros, similarly to European series, and cons: “still requires adaptation to new technology and shows limited maneuverability, with no current cost-effectiveness data available”.

As well, Finocchiaro et al. [33], with his multicenter, multi-surgeon study, aimed to define the learning curve and proficiency for SP-RARP. The results of 327 patients of three US centers (111 with transperitoneal and 216 with extraperitoneal approach) demonstrate that surgeons typically reach stable surgical performance, reducing operative time, after about 55 extraperitoneal cases, though estimated blood loss continues to vary independently by surgeon’s experiences. The absence of a plateau in the transperitoneal approach suggests different learning dynamics and possibly greater technical complexity. The study emphasizes that case complexity increases with experience. They concluded that SP-RARP is a safe and feasible technique, but achieving proficiency, especially in the extraperitoneal approach, but no proficiency plateau was observed for the transperitoneal route, indicating ongoing variability.

Ferguson et al. [34] reported the largest comparative analysis to date, assessing SP transperineal (TP) and transvesical (TV) radical prostatectomy in patients with a history of extensive abdominal surgery. Among 51 patients (18 TP and 33 TV), all procedures were completed without conversion or bowel injury. The SP-TV approach demonstrated a significantly shorter operative time, reduced opioid requirement, and shorter length of stay (mean 5.6 h vs. 22 h for TP), with superior continence recovery at one year (92% vs. 67%).

Katsimperis et al. [35] conducted a meta-analysis including 39 studies encompassing 2875 patients about radical prostatectomy, partial and radical nephrectomy, and radical cystectomy comparing SP vs. MP approach, demonstrating several advantages of SPS. Specifically, SP procedures were associated with lower estimated blood loss (mean difference −45 mL, *p* = 0.02) and shorter hospital stay (mean difference −0.6 days, *p* = 0.01) compared with MP surgery. No significant differences were observed in operative time (*p* = 0.09), major complication rate (Clavien ≥ III, *p* = 0.62), or positive surgical margins (*p* = 0.78). Overall, it confirmed the results of other studies where SPrs compare oncological safety to MPrs, providing statistically perioperative benefits.

These findings are confirmed and reinforced also in reconstructive urological procedures by a meta-analysis that reported lower estimated blood loss and better cosmetic results with SP, while maintaining comparable success and complication rates [36].

Celotto et al. [37] drafted a systematic review highlighting SPRS applications in general surgery in the US. Early evidence suggested shorter operative times, reduced pain, and faster recovery in colorectal, hepatobiliary, and abdominal wall procedures, although larger prospective studies are still required.

Furthermore, Kossenas has extended the general surgery with the study of single-port robotic cholecystectomy (SPRC) in patients with BMI > 25 kg/m^2^, comparing it with the same surgery with single-incision laparoscopic cholecystectomy (SILC). The study included 734 patients (365 SPRC and 369 SILC) where the operative duration was significantly longer for SPRC than SILC due to the learning curve, but intraoperative blood loss and complications and the length of hospitalization were the same. These results concluded that SPRC is safe and feasible also in overweight or obese patients [38].

In the thoracic surgery, Zervos et al. [39], with a multicenter prospective feasibility study of pulmonary lobectomy with SP via a 4 cm subcostal uniportal approach in nineteen patients, and Marshal et al. [40], with a prospective multicenter study of thymectomy SP approach with subxiphoid incision in thirteen patients, obtain similar results such as an estimated lower blood loss, less pain, and less hospital stay, but longer procedure time due to the learning curve. In summary, Zervos et al. [39] state using the SP robotic system is safe and feasible with a complication profile within the range reported for other uniportal and multi-portal robotic approaches. That said, Marshall et al. [40] emphasized that further clinical studies are needed to determine whether the subxiphoid SP approach provides meaningful clinical advantages over conventional multi-port intercostal robotic techniques.

The gynecologic field has received validation from both systematic and clinical reports which demonstrate that SP-assisted techniques are safe and feasible for use in transvaginal and natural-orifice procedures. Arcieri et al. [41] performed a full systematic review to assess da Vinci SP system usage in gynecologic procedures which demonstrated successful technical results with minimal complications and fast recovery times for both benign and oncologic procedures. SPRS provides superior instrument articulation and a single access point, which leads to an improved pelvic space access than multi-port systems for complex hysterectomies and pelvic staging procedures. Guan et al. [26] reported a series case of Robot-Assisted Single-Port vaginal Natural Orifice Transluminal Endoscopic Surgery (RSP-vNOTES) and showed that the procedure was technically possible and resulted in good cosmetic outcomes and fast recovery without any major complications or need for conversion. SPRS is going through a natural progression toward scarless gynecologic surgery which combines robotic precision with minimally invasive techniques.

The SP platform has also been proven successful for gender-affirming surgery. Dy et al. [42] reported peritoneal flap vaginoplasty procedures conducted with da Vinci SP and Xi systems to show that SP reduced surgical time without affecting postoperative results or complication rates. The single-port design improved ergonomics while allowing two surgeons to work together through the abdominal–perineal approach which enhanced their ability to access and coordinate within the limited pelvic area, asserting its advantages on working in restricted spaces that multi-arm platforms cannot reach.

These findings presented in this analysis confirm the early stages, yet expanding across different surgical specialties, of European experience. The versatility of the SP approach enables surgeons to perform various interdisciplinary surgeries, reducing the number of incisions and improving ergonomic comfort for the operator as stated by Nguyen et al. [43].

Overall, US centers benefit from earlier access to the platform, larger case series, and structured prospective data collection, which have enabled the definition of standardized techniques (e.g., transvesical, extraperitoneal, and retroperitoneal approaches) and the reporting of functional outcomes. In contrast, European experience remains limited, mostly consisting of small case series and video reports. Major gaps include the absence of multicentric registries, cost-effectiveness analyses, and long-term follow-up data. Moreover, restricted device availability and the late EMA approval in 2024 have delayed widespread adoption across European centers.

In summary, while early European reports confirm the technical feasibility and safety of SP surgery, the United States currently leads in clinical validation, procedural diversification, and standardization. Bridging this gap will require collaborative registries, training programs, and broader access to the SP system across European institutions.

Most of the European studies are only case report technical notes with few case series and only a comparative study reported faster docking time and a steady improving learning curve, no matter the steepness, with better operation time procedure after procedure.

The slower uptake of the da Vinci SP system in Europe appears to reflect a combination of differences in regulatory, logistical, and economic factors with respect to the US. The CE-Mark was granted in early 2024, meaning that broad clinical rollout only began thereafter. In addition, the manufacturer adopted a selective rollout strategy for European countries, which likely contributed to fewer high-volume centers accumulating experience. This contrasts with the US, where FDA clearance was obtained earlier and more widespread use in centers of excellence allowed for faster case accumulation and comparative analyses.

However, these promising European findings make us hope that soon we will be able to match U.S experience.

## 5. Limitations

Most of the included studies were single-center, retrospective, and descriptive, leading to limited statistical power and heterogeneity. Quantitative synthesis was not feasible due to variability in outcomes and methodologies. Publication bias cannot be excluded.

In line with evidence-based surgery principles, future prospective, multicentric, and comparative studies will be essential to generate higher-quality data. Once such evidence becomes available, a meta-analysis will be warranted to quantitatively validate the present findings. Also, direct comparison with US studies was not feasible.

This context highlights the need for continued collaboration among European centers to generate mature data and enable reliable cross-continental comparisons.

## 6. Conclusions

At present, Europe appears to be lagging behind the United States, mainly due to the still limited number of available studies. However, the results published so far are promising and suggest that comparable levels of effectiveness and adoption may be achieved. An additional limitation is the scarcity of consolidated evidence, likely related to ongoing or yet unpublished studies considering that approximately over 40 da Vinci Single-Port Systems are installed and operative at this point in Europe [44], and lacks different kinds of approach.

The recent propensity-matched analysis by Vizza et al. [15] (2025) represents the first comparative European experience directly evaluating the SP vs. MP robotic systems in a homogeneous oncologic cohort. It provides an initial framework for future prospective validation. Since the da Vinci SP platform obtained CE-Mark approval and became available for clinical use only in early 2024, its implementation in Europe is still at an initial stage. The limited time elapsed justifies the current paucity of prospective or multicenter studies; however, the growing experience of high-volume centers is expected to progressively strengthen the European evidence supporting SP robotic surgery.

The heterogeneity of published experiences reflects the growing interest of various surgical specialties and the need for shared training and data collection protocols.

Prospective, multicenter studies directly comparing SP surgery with MP surgery will be needed to more precisely define the clinical, ergonomic, and cost advantages of the SP system.

In line with the PRISMA recommendations and the principles of evidence-based surgery, a future meta-analysis will be necessary as soon as large prospective and comparative studies are published, capable of providing solid quantitative data and direct comparison with multi-port robotic surgery.

## Figures and Tables

**Table 1:** European experience with the da Vinci SP system—summary of key studies (ordered as cited in the manuscript).

Author (Year)	Country/Institution (Setting)	Surgical Field/Procedure	Study Design/N	Patient Position	Access/Incision Site	Robotic System & Configuration	Key Technical Notes (Arms and Camera Setup)	Main Outcomes
Izzo et al. [8] 2025	Italy—IRCCS Istituto Nazionale Tumori Fondazione G. Pascale, Naples	Urology—SP Nephroureterectomy	Technical report/8	Supine with flank bump, left arm abducted	6 cm lower abdominal (McBurney)	da Vinci SP, ‘camera-below’	Scissors R/Bipolar L/Cadiere A/Camera below	OR 108 min; EBL 130 mL; discharge < 24 h
Altez-Fernandez et al. [9] 2025	Spain—Complejo Hospitalario Universitario de A Coruña	Urology—SP Retroperitoneal Partial Nephrectomy	Case series/10	Lateral decubitus (flexed trunk)	4 cm retroperitoneal	da Vinci SP	30° up camera; 3 arms; no assistant port	Console 84 min; EBL 71 mL; VAS 2.5
Perdonà et al. [10] 2025a	Italy—IRCCS Pascale Foundation, Naples	Urology—SP Bilateral Inguinal Lymph Node Dissection	Case report	Supine, legs abducted	Single midline incision	da Vinci SP	TilePro 3-D guidance through midline port	Adequate nodal yield; low pain
Perdonà et al. [11] 2025b	Italy—IRCCS Pascale Foundation, Naples	Urology—SP Retroperitoneal Lymph Node Dissection (RPLND)	Case report	Supine	2.5 cm McBurney	da Vinci SP, ‘camera-below’	3-instrument triangulation	OR 79 min; no complications; discharge POD 2
Brassetti et al. [12] 2025	Italy—Fondazione Policlinico Universitario A. Gemelli, Rome	Urology—SP Antegrade Robotic Lymphadenectomy	Case series/10	Supine, thighs parted, 15° Trendelenburg	3.5 cm infra-umbilical	da Vinci SP	Endoscope 6 o’clock; cobra mode	OR 197 min; negligible EBL; cosmetic advantage
Chauleur et al. [13] 2025	France—CHU Saint-Étienne University Hospital Center	Gynecology—SP Retroperitoneal Para-aortic Lymphadenectomy	Video abstract/1	Supine	Infra-mesenteric retroperitoneal single port	da Vinci SP	Balloon space creation; 3 SP arms; optional accessory trocar	OR 58 min; same-day discharge; low pain; minimal scarring
Vizza et al. [14] 2025a	Italy—IRCCS Regina Elena National Cancer Institute, Rome	Gynecology—SP Hysterectomy (R-SPH)	Retrospective series	Dorsal lithotomy, steep Trendelenburg	2.5 cm trans-umbilical	da Vinci SP	Coaxial 3-arm + endoscope setup	EBL < 20 mL; LOS 1–2 days
Vizza et al. [15] 2025b	Italy—IRCCS Regina Elena National Cancer Institute, Rome	Gynecology—SP vs. MP Hysterectomy	Propensity-matched study/354 (50 SP, 50 MP)	Dorsal lithotomy, steep Trendelenburg	2.5 cm trans-umbilical SP vs. multi-port setup	da Vinci SP vs. da Vinci Xi	Coaxial 3-arm + endoscope setup	Docking time ↓ (6 vs. 11 min), *p* < 0.001
Chiofalo et al. [16] 2025	Italy—IRCCS Regina Elena National Cancer Institute, Rome	Gynecology—SP Ovarian Cancer Staging	Case series/7	Supine, low Trendelenburg	3 cm trans-umbilical	da Vinci SP	Maryland bipolar + monopolar scissors	EBL 10 mL; no grade ≥ II events; LOS 3 days
Vicente et al. [17] 2025	Spain—HM Sanchinarro University Hospital, Madrid	General Surgery—SP Cholecystectomy	Technical note/1	Supine, reverse Trendelenburg	2.5 cm umbilical	da Vinci SP	3 arms through single cannula, 0° camera	Dock 10 min; OR 38 min; VAS < 3
Hadzijusufovic et al. [18] 2025	Germany—University Medical Center Mainz	Upper GI—SP Subcostal Esophagectomy (SC-RAMIE)	Technical report	Left lateral → semi-prone	4 cm subcostal	da Vinci SP	Bipolar (1), Retractor (2), Scissors (3), 0° camera	Complete esophagectomy; low pain
Hadzijusufovic et al. [19] 2024	Germany—University Medical Center Mainz	Upper GI—SP Cervical Esophagectomy (RACE)	Technical report	Supine, neck extended, head right	3 cm left cervical	da Vinci SP	30° cobra; 3-arm triangulation	Nerve preservation; no thoracic access
Hipp et al. [20] 2025	Germany—LMU Hospital, Munich	Abdominal Wall—SP Extended Extraperitoneal Plasty (eTEP)	Technical note	Supine	Single midline incision	da Vinci SP	Flexible scope; 3 arms	Feasible; no recurrence; no complications
Seifen et al. [21] 2025	Germany—University Hospital Cologne	ENT—SP Transoral Tonsillectomy	Case report	Supine, head reclined	Transoral	da Vinci SP	Port 10 cm above mouth; monopolar + bipolar	Dock 8 min; OR 37 min; no complications

Abbreviations: LAA—Low Anterior Access; MP—Multi-Port; POD—Postoperative Day; SPARL—Single-Port Antegrade Robotic Lymphadenectomy; FReTEP—Fully Robotic Extended Totally Extraperitoneal Plasty.

## Data Availability

All data analyzed in this review are contained within the cited articles.

## Abbreviations

The following abbreviations are used in this manuscript:

SP	Single-Port
MP	Multi-Port
SPRS	Single-Port Robotic Surgery
RANU	Robot-Assisted Nephroureterectomy
RAPN	Robot-Assisted Partial Nephrectomy
ILND	Inguinal Lymph Node Dissection
RPLND	Retroperitoneal Lymph Node Dissection
SPARL	Single-Port Antegrade Robotic Lymphadenectomy
LAA	Low Anterior Access
eTEP	Extended Totally Extraperitoneal Plasty
FReTEP	Fully Robotic Extended Totally Extraperitoneal Plasty
EMA	European Medicines Agency
FDA	Food and Drug Administration
ROSI	Remotely Operated Suction Irrigation System
AirSeal^®^	Valveless insufflation system (ConMed, Utica, NY, USA)
vNOTES	Vaginal Natural Orifice Transluminal Endoscopic Surgery
US	United States
UTUC	Upper Tract Urothelial Carcinoma
POD	Postoperative Day
3DHD	Three-Dimensional High Definition
TORS	Transoral Robotic Surgery
RSP-vNOTES	Robotic Single-Port vaginal Natural Orifice Transluminal Endoscopic Surgery
